# Effect of pretreatment lab abnormalities on the time-to-treatment discontinuation and overall survival of metastatic breast cancer patients receiving CDK 4/6i, PI3Ki, and/or mTORi

**DOI:** 10.1007/s10549-025-07751-1

**Published:** 2025-06-18

**Authors:** Jeffrey Franks, Andres Azuero, Kelly Kenzik, Nusrat Jahan, Mackenzie Fowler, Russell Griffin, Gabrielle Rocque

**Affiliations:** 1 Division of Hematology and Oncology, Department of Medicine, University of Alabama at Birmingham, Birmingham, AL USA; 2 Department of Epidemiology, University of Alabama at Birmingham, Birmingham, AL USA; 3 O’Neal Comprehensive Cancer Center, Birmingham, AL USA; 4 School of Nursing, University of Alabama at Birmingham, Birmingham, AL USA; 5 Department of Surgery, Chobanian and Avedisian School of Medicine, Boston Medical Center, Boston University, Boston, MA USA

**Keywords:** Targeted therapies, Metastatic breast cancer, Comorbidities, Survival

## Abstract

**Purpose:**

Metastatic breast cancer (MBC) randomized controlled trials (RCTs) enroll healthier patients than the general population; however, many women have a lab abnormality at the time of their diagnosis, RCTs inadequately represent this patient population. To better understand this population, this study estimated time-to-treatment discontinuation (TTD) and overall survival (OS) for patients with and without lab abnormalities receiving a targeted therapy for MBC.

**Methods:**

This retrospective study used the nationwide, de-identified electronic health record-derived Flatiron Health database to include women with hormone receptor-positive, Human epidermal growth factor receptor 2- negative MBC with receipt of a targeted therapy between 2011 and 2020. Abnormalities were defined by common exclusionary cut-offs in targeted therapy clinical trials. TTD was defined as time from treatment initiation to the first occurrence of either treatment change or death. The secondary outcome was OS defined as time from treatment initiation to death from any cause. Accelerated failure time models estimating the survival time ratio, predicted mean survival time differences, and 95% confidence intervals (CIs) were used for the association between lab abnormalities and TTD or OS.

**Results:**

Among patients with receipt of a CDK 4/6 inhibitor, patients with at least one lab abnormality had a 33% shorter TTD (MR 0.67; 95% CI 0.59, 0.68) and 25% shorter OS (MR 0.75; 95% CI 0.70, 0.81) than those with no lab abnormalities. More modest differences were seen in TTD and OS for patients with receipt of Everolimus or Alpelisib. Patients saw the largest impact with liver abnormalities with 25% to 45% shorter TTD and 38% to 66% shorter OS across the treatment types. Interestingly, while only patients with receipt of a CDK 4/6i saw significantly shorter TTD for patients with blood abnormalities, patients with receipt of Alpelisib additionally saw shorter OS.

**Conclusion:**

Patients with lab abnormalities saw significantly lower TTD and OS than those without abnormalities. Patients with liver abnormalities saw significantly shorter TTD and OS across all treatments likely driving this association. More real-world studies of patients with lab abnormalities are needed to empower oncologists when making treatment decisions in high-risk populations, to discuss prognosis and to inform RCT eligibility criteria.

## Background

Randomized control trials (RCTs) frequently enroll healthier patients with less abnormalities than the general population [1]. In an analysis of 522 clinical trials for breast cancer, Szlezinger et al. reported that [2] 77% of trials had at least one strict comorbidity exclusion criteria [3]. Gidwani et al. found in early-stage breast cancer (EBC) trials that approximately half of patients would be unrepresented due to their lab abnormalties [4]. This is problematic because the Food and Drug Administration (FDA) relies on RCTs to approve new cancer treatments. However, given that up to 42% of women have a comorbidity at the time of their breast cancer diagnosis, many breast cancer RCTs may inadequately represent the real-world patient population [5], limiting larger scale applicability of results regarding patient safety, treatment decision-making, and prognosis.

Inferences for the general breast cancer population often rely on observational or cohort studies. In one study, patients with EBC and concurrent lab abnormalities receiving chemotherapy had a nearly threefold increased risk of death after controlling for their cancer characteristics and other demographics [4]. However, the study was limited to EBC where prognosis is often good and duration of treatment is limited [6]. Few studies exist focusing on the lab abnormality and treatment relationship for metastatic breast cancer (MBC), which in contrast to EBC is not curable and is often the patients’ cause of death. EBC is often treated by anthracycline and taxane-based chemotherapies, and it is unclear if patients with lab abnormalities receiving novel targeted therapies offered in the MBC setting will have the same associations as they often have more unique but fewer side effect profiles [6, 7].

For those diagnosed with MBC, targeted therapies, such as Cyclin-dependent kinase 4/6 inhibitors (CDK4/6i), alpelisib, and everolimus, have become the standard of care for treating hormone receptor-positive (HR +), human epidermal growth factor receptor 2-negative (HER2-) [8–10]. These trials too had laboratory criteria limiting the information that oncologists have for making informed treatment decisions. Therefore, the purpose of this study was to estimate the time-to-treatment discontinuation (TTD) and overall survival for patients with and without lab abnormalities receiving a targeted therapy for MBC.

## Methods

### Study design and patient population

This retrospective cohort study used the nationwide, electronic health record-derived Flatiron Health database to include women diagnosed with metastatic breast cancer between 2015 and 2022. Flatiron Health is a longitudinal database composed of de-identified patient-level structured and unstructured data curated from approximately 280 US oncology care sites (~ 800 sites of care), including community cancer practices and academic medical centers [11]. Patients with HR+ HER2− MBC from the Flatiron Health MBC database were included in our cohort. We included women receiving at least one of three targeted therapies, CDK4/6i (Palbociclib, Abemaciclib, or Ribociclib), everolimus, or alpelisib and who had complete labs for platelets, neutrophils, lymphocytes, hemoglobin, glucose, hemoglobin A1c, creatinine, alanine transaminase (ALT), and aspartate transaminase (AST). Additionally, patients included were required to be on an FDA approved regimen (i.e., have concurrent endocrine therapy with CDK 4/6i, alpelisib, or everolimus). We excluded patients who were male, aged less than 18 years old, had missing cancer subtype or treatment, and who had targeted therapy without hormone therapy. This study was approved by the University of Alabama at Birmingham Internal Review Board.

### Outcomes: time-to-treatment discontinuation and overall survival

The primary outcome for this study is time-to-treatment discontinuation (TTD). The TTD was defined as time from first targeted therapy treatment initiation to the first occurrence of either treatment switch due to any reason (progression or toxicities) or death. The secondary outcome was overall survival (OS) defined as time from first targeted therapy treatment initiation to death from any cause. Death was recorded in the Flatiron Health database by aggregating structured and unstructured EHR records, Social Security Death Index, and obituaries [12].

### Exposure: lab abnormalities

The primary exposure of interest was the presence of a lab abnormality prior to treatment initiation. Lab tests included Platelet count, Neutrophil count, Lymphocyte count, Hemoglobin, Glucose, Hemoglobin A1c, Creatinine, ALT, AST, and bilirubin. Lab abnormalities were determined by common exclusionary cut-offs in targeted therapy clinical trials (Appendix Table 4) [8, 13, 14]. A glucose cut-off of > 199 mg/dL was used to supplement hemoglobin A1c as it was not well captured in the dataset. A binary value for lab abnormalities prior to treatment initiation was determined by the presence of at least one lab abnormality within 30 days prior to treatment initiation vs. no lab abnormalities. Secondary analysis included categorizing lab tests into organ systems. Platelets, neutrophils, lymphocytes, and hemoglobin were grouped into the hematological category; Glucose and Hemoglobin A1c were grouped as the glucose metabolism category; Creatinine was considered the kidney category; and ALT, AST, and bilirubin were grouped as the liver category.

### Patient characteristics

Patients’ age was calculated at the date of their MBC diagnosis. Race and ethnicity were categorized as White, Black, other, or Not documented. Other race and ethnicity included Hispanic or Latino, Asian, American Indian, Alaskan Native, and Pacific Islanders; these races and ethnicities were combined due to small sample sizes in some categories in the dataset.

### Clinical characteristics

Clinical characteristics were measured at MBC diagnosis. Cancer subtype was determined through hormone receptor and human epidermal growth factor receptor 2 biomarker tests. The site of metastasis was categorized as visceral, bone only, or lymph nodes only. Patients with at least one visceral site were considered visceral, those without visceral sites but with at least one bone site were considered bone only, and those with only lymph node sites were considered lymph node only disease.

### Treatment characterization

Palbociclib, Ribociclib, and Abemaciclib were grouped as CDK 4/6i. Treatments were identified using generic drug names recorded as either ordered or administered within the Flatiron Health database. Concurrent treatments were categorized as hormone only or hormone and chemotherapy. Finally, the total lines of therapy for MBC prior to the treatment of interest was calculated.

### Statistical analysis

Descriptive analyses included medians and interquartile ranges (IQRs) for continuous variables or frequencies and percentages for categorical variables. Parametric accelerated failure time (AFT) models estimating the survival time ratio, predicted mean survival time differences, and 95% confidence intervals (CIs) were used for the association between lab abnormalities and TTD among patients receiving one of our three treatments of interest. The AFT models estimate the geometric mean as opposed to the arithmetic mean common in other models but will be referred to as the mean. Analyses consisted of at least one lab abnormality vs. none and stratified by organ system (blood, glucose metabolism, kidney, and liver). Additional analysis using multivariable AFT models assessed the association between lab abnormalities and overall survival. Sensitivity analysis were performed to assess TTD and OS by individual CDK 4/6i treatments (Palbociclib, Abemaciclib, or Ribociclib). All models were adjusted for age at MBC diagnosis, race/ethnicity, site of metastasis, concurrent treatment, and line of therapy. Goodness of fit among different assumed survival distributions was assessed for all AFT models using the Bayesian information criterion. A Gamma distribution was found to have the best fit for all models. A *p*-value of < 0.05 was considered statistically significant and all statistical analyses were performed using SAS version 9.4 (SAS, Cary, NC).

## Results

### Patient characteristics

A total of 6,007 women with HR+ HER2− MBC with receipt of at least one CDK4/6i, everolimus, or alpelisib during their treatment course were eligible and included in the analysis. Demographics and clinical characteristics of study participants overall and by treatment regimens are shown in Table 1. The median age of patients was 63 (IQR 55–72) with the majority being White (68%), followed by Black (10%). The most common location of metastasis was Visceral (74%). Patients with receipt of a CDK 4/6i (*n* = 5239) had a median of 0 prior lines of therapy (IQR 0–1) with 2% receiving both concurrent hormone therapy and chemotherapy. Patients with receipt of everolimus (*n* = 1585) had a median of 2 (IQR 1–3) prior lines of therapy with 3% receiving concurrent hormone treatment and chemotherapy. Finally, patients with receipt of alpelisib (*n* = 601) had a median of 3 (IQR 1–4) prior lines of therapy with 4% receiving concurrent hormone treatment and chemotherapy. Overall, 69% of patients had no lab abnormalities, 30% had 1 to 2 lab abnormalities, and 1% had 3 or more lab abnormalities within 30 days prior to treatment initiation.

**Table 1:** Patient and clinical characteristics by treatment

Characteristics	Overall^a^	CDK 4/6i	Everolimus	Alpelisib
	n = 6007 n (%)	n = 5239 n (%)	n = 1585 n (%)	n = 601 n (%)
Age at MBC diagnosis (years)				
Median, IQR	63 (55–72)	64 (56–73)	62 (55–70)	62 (54–69)
Race and ethnicity				
White	4114 (68)	3576 (68)	1091 (69)	402 (67)
Black	581 (10)	520 (10)	144 (9)	45 (7)
Asian	121 (2)	106 (2)	35 (2)	22(4)
Hispanic	319 (5)	275 (5)	96 (6)	38 (6)
Other race	470 (8)	399 (8)	140 (9)	52 (9)
Unknown	402 (7)	363 (7)	79 (5)	42 (7)
Site of metastasis^b^				
Visceral	4415 (74)	3768 (72)	1346 (85)	520 (87)
Bone	1508 (25)	1395 (26)	232 (15)	78 (13)
Lymph node	74 (1)	66 (1)	6 (0.4)	3 (1)
Prior lines of therapy				
Median, IQR	–	0 (0–1)	2 (1–3)	3 (1–4)
Concurrent treatment^c^				
Hormone only	–	5131 (98)	1528 (85)	512 (85)
Hormone and chemotherapy	–	108 (2)	57 (3)	25 (4)
Lab abnormalities				
None	4179 (69)	3660 (70)	1086 (60)	326 (54)
1 to 2	1761 (30)	1524 (29)	686 (38)	258 (43)
3 or more	67 (1)	55 (1)	32 (2)	17 (3)

*CDK 4/6i* Cyclin-dependent kinase 4/6 inhibitors, *MBC* metastatic breast cancer, *IQR* interquartile range

^a^If more than one targeted therapy is used in a line then the first therapy was used for overall results

^b^Unknown site of metastasis not shown due to small cell size

^c^alpelisib allowed for non-hormone concurrent regimens; *n* = 60 received no concurrent treatments and *n* = 4 patients received concurrent chemotherapy only

### CDK 4/6 inhibitors

Table 2 shows results of TTD after adjusting for age, race/ethnicity, site of metastasis, concurrent treatment, and number of prior lines of therapy. Patients with at least one lab abnormality had 33% (MR 0.67; 95% CI 0.59, 0.68) lower TTD than those with no lab abnormalities. On average, the predicted TTD for those with no lab abnormalities was 8 months longer than those with lab abnormalities. The TTD differed by abnormalities in organ system where those with liver function abnormalities had 44% lower TTD, those with blood abnormalities had 31% lower TTD, those with kidney abnormalities had 21% lower TTD, and those with pancreatic abnormalities had 13% lower TTD compared to those without. Sensitivity analysis assessed individual CDK 4/6i treatments, similar results were seen across the three CDK 4/6i as to the combined analysis (Appendix Table 5). However, TTD was much lower for patients receiving Abemaciclib with kidney abnormalities (MR 0.58; 95% CI 0.39, 0.87), compared to those receiving Palbociclib (MR 0.86; 95% CI 0.69, 1.07) or Ribociclib (MR, 1.29; 95% CI 0.68, 2.45). Similar differences in survival were seen when assessed as overall survival (Table 3) where those with at least one abnormality had a 25% (MR 0.75; 95% CI 0.70, 0.81) lower overall survival than those with no lab abnormalities. OS differed by organ system with the largest differences seen in liver abnormalities where those with the abnormality had 44% (MR 0.56; 95% CI 0.48, 0.64) lower overall survival compared to those without. Among the other organ systems, those with glucose metabolism abnormalities had 15% (MR 0.85; 95% CI 0.75, 0.97) lower overall survival, and those with blood abnormalities had 9% (MR 0.91; 95% CI 0.84, 0.99) lower overall survival. Kidney abnormalities were not statistically associated with decreased survival.

**Table 2:** Time-to-treatment discontinuation comparing none vs. at least one lab abnormality overall and by organ system

	CDK 4/6i *n* = 5239		Everolimus *n* = 1585		Alpelisib *n* = 601	
	Mean ratio (95% CI)	Mean survival months (95% CI)	Mean ratio (95% CI)	Mean survival months (95% CI)	Mean ratio (95% CI)	Mean survival months (95% CI)
Overall						
None	Ref	21.7 (16.4, 28.6)	Ref	7.2 (4.5, 11.5)	Ref	5.9 (3.6, 9.6)
At least one	0.67 (0.59, 0.68)	13.7 (10.3, 18.2)	0.90 (0.83, 0.98)	6.4 (4.0, 10.4)	0.75 (0.64, 0.87)	4.4 (2.7, 7.2)
Blood						
None	Ref	20.7 (15.6, 27.3)	Ref	6.9 (4.4, 11.2)	Ref	5.42 (3.51, 8.38)
At least one	0.69 (0.63, 0.75)	14.2 (10.6, 18.9)	0.95 (0.86, 1.05)	6.6 (4.1, 10.7)	0.87 (0.74, 1.03)	4.75 (3.03, 7.42)
Glucose metabolism						
None	Ref	19.3 (14.6, 25.6)	Ref	8.1 (5.02, 13.2)	Ref	5.41 (3.53, 8.28)
At least one	0.87 (0.76, 0.99)	16.8 (12.3, 22.8)	0.92 (0.78, 1.09)	7.5 (4.5, 12.4)	0.74 (0.57, 0.97)	4.01 (2.46, 6.53)
Kidney						
None	Ref	19.1 (14.4, 24.9)	Ref	6.9 (4.4, 11.1)	Ref	5.22 (3.37, 9.65)
At least one	0.79 (0.65, 0.97)	15.2 (10.7, 21.4)	0.92 (0.77, 1.08)	6.1 (3.6, 10.3)	0.98 (0.61, 1.58)	6.11 (2.70, 9.65)
Liver						
None	Ref	19.3 (14.6, 25.5)	Ref	7.0 (4.4, 11.2)	Ref	5.20 (3.38, 8.01)
At least one	0.56 (0.48, 0.64)	10.8 (7.9, 14.7)	0.75 (0.64, 0.89)	5.3 (3.2, 8.7)	0.61 (0.47, 0.79)	3.17 (1.92, 5.23)

*CDK 4/6i* CDK 4/6 inhibitor, *CI* confidence intervals

Models were adjusted for age at metastatic diagnosis, race/ethnicity, site of metastasis, concurrent treatment, and line of therapy

**Table 3:** Overall survival comparing none vs. at least one comorbidity overall and by organ system

	CDK 4/6i *n* = 5239		Everolimus *n* = 1585		Alpelisib *n* = 601	
	Mean ratio (95% CI)	Mean survival months (95% CI)	Mean ratio (95% CI)	Mean survival months (95% CI)	Mean ratio (95% CI)	Mean survival months (95% CI)
Overall						
None	Ref	62.5 (41.1, 94.9)	Ref	15.8 (13.2, 18.9)	Ref	15.7 (9.9, 24.8)
At least one	.75 (0.70,.81)	46.7 (30.7, 71.6)	0.88 (0.76, 0.98)	13.9 (11.5, 16.8)	0.63 (0.51, 0.78)	9.8 (6.2, 15.7)
Blood						
None	Ref	60.7 (39.6, 93.0)	Ref	14.6 (11.9, 17.9)	Ref	15.1 (9.2, 24.7)
At least one	.91 (.84,.99)	55.2 (35.8, 85.1)	.96 (0.85, 1.09)	15.2 (12.7, 18.1)	0.69 (0.55, 0.87)	10.5 (6.2, 17.8)
Glucose metabolism						
None	Ref	60.2 (39.3, 92.2)	Ref	16.6 (12.7, 21.6)	Ref	13.8 (8.3, 22.8)
At least one	0.85 (0.75, 0.97)	51.4 (33.0, 80.2)	1.12 (0.89, 1.40)	14.8 (12.4, 17.8)	1.26 (.87, 1.83)	17.4 (9.7, 31.3)
Kidney						
None	Ref	59.9 (39.1, 91.8)	Ref	15.1 (10.5, 21.7)	Ref	14.2 (8.6, 23.5)
At least one	0.86 (0.70, 1.04)	51.4 (32.1, 82.2)	1.00 (0.72, 1.39)	15.0 (12.6, 17.9)	1.11 (0.56, 2.2)	15.8 (7.0, 35.9)
Liver						
None	Ref	58.4 (38.5, 88.4)	Ref	15.2 (12.7,18.2)	Ref	13.5 (8.4, 21.7)
At least one	0.56 (0.48, 0.64)	32.4 (20.9, 50.4)	0.62 (0.50, 0.77)	9.4 (7.2, 12.4)	0.34 (0.24, 0.48)	4.6 (2.6, 8.2)

*CDK 4/6i* CDK 4/6 inhibitor, *CI* confidence intervals

Models were adjusted for age at metastatic diagnosis, race/ethnicity, site of metastasis, concurrent treatment, and line of therapy

### Everolimus

In our sample of patients with receipt of everolimus, more modest differences were seen in TTD. After adjusting for covariates, patients with at least one lab abnormality had 10% (MR 0.90; 95% CI 0.83, 0.98) lower TTD than those with no lab abnormalities. The predicted TTD, on average, for those with no lab abnormalities was 0.8 months longer than those who had lab abnormalities. This finding was driven by liver abnormalities, where patients had 25% (MR 0.75; 95% CI 0.64, 0.89) lower TTD than those without; no other organ systems had statistically significant differences. Overall survival had similar results with 12% (MR 0.88; 95% CI 0.76, 0.98) lower OS for patients with at least one abnormality compared to those without. Similarly, only those with liver abnormalities saw significant OS differences with 38% (MR 0.62; 95% CI 0.50, 0.77) lower OS for patients with vs. without the abnormalities.

### Alpelisib

Among patients with receipt of alpelisib, those with any lab abnormalities had 25% (MR 0.75; 95% CI 0.64, 0.87) lower TTD than those without any abnormalities. On average, patients without any lab abnormalities had 1.5 months longer survival than those with abnormalities. Similar to the CDK 4/6i and everolimus analyses, liver abnormalities had the largest difference with 39% (MR 0.61; 95% CI 0.47, 0.79) lower survival for those with vs. without abnormalities. However, glucose metabolism abnormalities were also different with those with glucose metabolism abnormalities experiencing 26% (MR 0.74; 95% CI 0.57, 0.97) lower survival than those without, but blood and kidney abnormalities saw no differences. For OS, those with any lab abnormalities had 37% (MR 0.63; 95% CI 0.51, 0.78) lower survival than those without. When stratified by organ system, patients with liver abnormalities had 66% (MR 0.34; 95% CI 0.24, 0.48) lower survival than those without, and in contrast to TTD, those with blood abnormalities had 31% (MR 0.69; 95% CI 0.55, 0.87) lower OS but no significant differences were seen with glucose metabolism or kidney abnormalities. Sensitivity analysis stratifying the CDK 4/6i by treatment again saw similar results in OS overall and by organ system (Appendix Table 6). Ribociclib saw the largest OS for patients with blood and glucose abnormalities compared to those without, while Abemaciclib saw lower differences for patients with kidney and liver abnormalities.

## Discussion

Patients with a pretreatment lab abnormality have lower TTD and lower OS than patients without a lab abnormality across three common targeted therapies for MBC. Additionally, we found that survival differences varied by the organ system encompassing the lab abnormality. For CDK4/6i, TTD was 13% lower for those with glucose metabolism abnormalities, 21% lower for kidney abnormalities, 31% lower for hematological abnormalities, and 44% lower for liver abnormalities. Trends were similar when stratifying individually assessing the CDK 4/6i. These findings are consistent with studies of common CDK4/6i toxicities where blood and liver abnormalities have been commonly reported [15], while kidney and pancreatic toxicities were less common [16, 17]. Interestingly, our findings by organ system varied by treatment where those with receipt of everolimus only saw significant liver abnormality differences while those with receipt of alpelisib only saw significant survival differences for both glucose metabolism and liver abnormalities. Though the results seem intuitive, few clinical trials assess outcome differences for patients with lab abnormalities limiting the amount of information oncologists have to make treatment decisions. Furthermore, the lack of impact of several lab abnormalities commonly used in trials suggests that a more nuanced approach to exclusion criteria is needed that considers more specifically the mechanism of the treatment and potential toxicities, rather than using traditional cut points. This may be particularly relevant in targeted therapy clinical trials, given the differences in mechanism and side effect profile observed with different medication classes.

Data on what to expect for patients with lab abnormalities is also critically important to physician–patient communication about risk. For patients with abnormalities, oncologists may erroneously apply prognosis based on data from patients without the lab abnormality of their patient, overestimating and providing unrealistic expectations. Further, in multidisciplinary team (MDT) meetings, where specialists gather to discuss and review patients, the lack of prognostic understanding may impact ability to generate consensus regarding treatment recommendations for patients with comorbidities [18]. The European Society for Medical Oncology (ESMO) guidelines provide some guidance in regards to patients with lab abnormalities by suggesting alternative treatments or treatment delays [19]; however, our analysis shows that patients with abnormalities are still routinely receiving these treatments within 30 days of a lab abnormality. Additionally, treatment delays can increase the risk of death and [20, 21] increase patient mental distress [22]; therefore, finding alternatives to treatment delays are crucial. More studies including patients with comorbidities or lab abnormalities are needed to empower oncologists and allow them to make data-driven decisions to ensure they are providing the most accurate care to their patients.

In addition to the need for more real-world studies assessing those with comorbidities, is the lack of representation of these patients within clinical trials. RCTs are considered the gold standard for the development of cancer treatments due to their excellent internal validity through the use of randomization and strict eligibility criteria and aim to demonstrate safety and efficacy of new treatments [23]. However, restricting the study population to the healthiest of individuals often reduces the generalizability of results to vulnerable populations [24]. The American Society of Clinical Oncology and Friends of Cancer Research (ASCO-Friends) released a call in 2017 to broaden RCT eligibility criteria and make trials more representative [25]. They state that some current exclusion cut-offs are unnecessary and some already have exceptions that could be broadened to other patients [26]. Broadening inclusion criteria is expected to greatly increase the representation of patients with comorbidities. However, it is also important to ensure the safety of these patients when doing so and understand the implications of including a more complex population in the outcomes of the RCTs. More real-world studies will facilitate the understanding of the impact of comorbidities in the survival of the patients and implications of broadening inclusion in RCTs. New analytic methods may be needed to help analyze the data with the inclusion of competing risks from patient’s concurrent lab abnormalities. Additionally, though the FDA has agreed that broadening eligibility criteria is necessary [27], it and other key bodies who approve drugs for market or evaluate trials will have to update processes to account for the inclusion of this new vulnerable population.

This study should be viewed in consideration of several limitations. All-cause mortality was used in place of cancer-specific mortality as specific causes of death were unavailable in our dataset. However, given that this cohort was comprised of metastatic patients it is likely that a large portion of mortality was due to their MBC. We did not utilize ICD codes to supplement our lab abnormalities as EHR ICD codes are currently lacking in their ability to identify comorbidities [28]. This means we were unable to account for several important criteria such as cardiovascular comorbidities. We were also unable to account for treatment toxicities; therefore, we are unsure what may have caused the patient to switch treatment. We utilized time-to-treatment discontinuation instead of progression-free survival as the reason for discontinuation was not available in our dataset. Finally, other residual confounding may remain as we were unable to account for all clinical and demographic characteristics such as genetic mutations.

## Conclusion

In a real-world sample of women with HR+ HER2− MBC treated with targeted therapies (e.g., CDK 4/6i, everolimus, or alpelisib) patients with lab abnormalities saw significantly lower TTD and overall survival than those without abnormalities. Differences in survival were seen by lab abnormalities in different organ system between the three treatments of interest demonstrating a need for data-driven RCT exclusionary cut-offs. Additionally, more real-world studies of patients with lab abnormalities are needed to not only empower oncologists when making treatment decisions and discussing prognosis, but also to help inform accurate eligibility criteria. Finally, key bodies need to develop processes to evaluate RCTs with the inclusion of a more complex population.

## Data Availability

The data that support the findings of this study have been originated by Flatiron Health, Inc. These de-identified data may be made available upon request, and are subject to a license agreement with Flatiron Health; interested researchers should contact < DataAccess@flatiron.com > to determine licensing terms.

## Appendix

See Tables

**Table 4:** Lab values used and their cut-offs based on common randomized clinical trial criteria

Organ system	Lab value	Lab value cut-off
Blood	Absolute neutrophil count	< 1.5 × 10^9^L (< 1500 cells/μL)
	Lymphocyte count	< 0.5 × 10^9^L (< 500/μL)
	Platelet	< 100 × 10^9^/L (< 100,000/μL)
	Hemoglobin	< 9 g/dL
Kidney	Creatinine clearance/Serum creatinine	Serum creatinine > 1.5 × ULN or estimated creatinine clearance < 60 mL/min
Liver	ALT and/or AST	> 3 × ULN (> 5 × ULN if liver metastases)
	Bilirubin	> 1.25 × ULN
Glucose metabolism	HbA1c	> 6.4
	Glucose	> 199 mg/dL

4,

**Table 5:** Time-to-treatment discontinuation comparing none vs. at least one lab abnormality overall and by organ system by CDK 4/6i type

	Palbociclib *n* = 4722		Ribociclib *n* = 515		Abemaciclib *n* = 1016	
	Mean ratio (95% CI)	Mean survival months (95% CI)	Mean ratio (95% CI)	Mean survival months (95% CI)	Mean ratio (95% CI)	Mean survival months (95% CI)
Overall						
None	Ref	19.5 (14.5, 26.2)	Ref	12.03 (7.8, 18.5)	Ref	12.2 (9.2, 16.2)
At least one	0.66 (0.62, 0.71)	12.9 (9.6, 17/5)	0.60 (0.48, 0.76)	7.2 (4.8, 11.0)	0.63 (0.53, 0.74)	7.6 (5.7, 10.3)
Blood						
None	Ref	19.0 (14.1, 25.6)	Ref	9.6 (6.2, 14.7)	Ref	11.2 (8.4, 14.9)
At least one	0.69 (0.63, 0.75)	13.2 (9.7, 17.8)	0.68 (0.52, 0.90)	6.6 (4.1, 10.4)	0.73 (0.61, 0.88)	8.2 (6.0, 11.2)
Glucose metabolism						
None	Ref	17.8 (13.2, 24.0)	Ref	10.0 (5.8, 13.8)	Ref	10.4 (7.8, 13.9)
At least one	0.92 (0.80, 1.06)	16.4 (11.9, 22.8)	0.84 (0.56, 1.29)	7.6 (4.4, 13.1)	0.95 (0.68, 1.32)	9.9 (6.5, 15.0)
Kidney						
None	Ref	17.7 (13.2, 23.9)	Ref	8.5 (5.5, 13.1)	Ref	10.9 (8.2, 14.4)
At least one	0.86 (0.69, 1.07)	15.3 (10.6, 22.0)	1.29 (0.68, 2.45)	11.0 (5.3, 22.9)	0.58 (0.39, 0.87)	6.4 (4.0, 10.1)
Liver						
None	Ref	17.8 (13.3, 24.0)	Ref	9.5 (6.2, 14.6)	Ref	10.6 (7.9, 14.0)
At least one	0.58 (0.50, 0.67)	10.4 (7.5, 14.5)	0.58 (0.36, 0.90)	5.5 (3.1, 9.6)	0.53 (0.39, 0.73)	5.6 (3.7, 8.5)

*CDK 4/6i* CDK 4/6 inhibitor, *CI* confidence intervals

Models were adjusted for age at metastatic diagnosis, race/ethnicity, site of metastasis, concurrent treatment, and line of therapy

5, and

**Table 6:** Overall survival comparing none vs. at least one lab abnormality overall and by organ system by CDK 4/6i type

	Palbociclib *n* = 4722		Ribociclib *n* = 515		Abemaciclib *n* = 1016	
	Mean ratio (95% CI)	Mean survival months (95% CI)	Mean ratio (95% CI)	Mean survival months (95% CI)	Mean ratio (95% CI)	Mean survival months (95% CI)
Overall						
None	Ref	41.1 (35.7, 47.3)	Ref	39.6 (26.3, 59.7)	Ref	40.4 (29.5, 55.5)
At least one	0.77 (0.71, 0.83)	31.6 (27.2, 36.7)	0.54 (0.41, 0.70)	21.3 (14.0, 32.4)	0.64 (0.52, 0.77)	25.7 (18.3, 36.0)
Blood						
None	Ref	39.9 (33.9, 44.8)	Ref	32.9 (22.2, 48.9)	Ref	37.9 (27.7, 51.7)
At least one	0.92 (0.84, 1.00)	35.8 (30.7, 41.7)	0.63 (0.47, 0.84)	20.8 (13.2, 32.9)	0.77 (0.62, 0.94)	29.0 (20.6, 40.9)
Glucose metabolism						
None	Ref	38.4 (33.5, 44.1)	Ref	31.7 (21.3, 47.1)	Ref	36.1 (26.5, 49.0)
At least one	0.92 (0.80, 1.05)	35.3 (29.2, 42.6)	0.73 (0.48, 1.12)	23.2 (13.4, 39.9)	0.76 (0.54, 1.08)	27.6 (17.6, 43.2)
Kidney						
None	Ref	38.4 (33.4, 44.0)	Ref	30.4 (20.5, 45.9)	Ref	35.7 (26.2, 48.5)
At least one	0.88 (0.71, 1.09)	33.9 (26.5, 43.4)	1.11 (0.59, 2.09)	33.8 (16.4, 69.6)	0.87 (0.56, 1.37)	31.1 (18.3, 53.0)
Liver						
None	Ref	38.1 (33.2, 43.8)	Ref	32.5 (21.7, 48.7)	Ref	35.4 (25.9, 48.4)
At least one	0.53 (0.46, 0.61)	20.3 (16.6, 24.8)	0.55 (0.35, 0.88)	18.0 (10.1, 32.0)	0.49 (0.34, 0.73)	17.5 (10.7, 28.6)

*CDK 4/6i* CDK 4/6 inhibitor, *CI* confidence intervals

Models were adjusted for age at metastatic diagnosis, race/ethnicity, site of metastasis, concurrent treatment, and line of therapy

6.
